# Risk Estimates and Risk Factors Related to Psychiatric Inpatient Suicide—An Overview

**DOI:** 10.3390/ijerph14030253

**Published:** 2017-03-02

**Authors:** Trine Madsen, Annette Erlangsen, Merete Nordentoft

**Affiliations:** 1 Danish Research Institute for Suicide Prevention, Copenhagen Mental Health center, 2900 Hellerup, Denmark; Annette.Erlangsen@regionh.dk (A.E.); merete.nordentoft@regionh.dk (M.N.); 2 Department of Mental Health, Bloomberg Johns Hopkins School of Public Health, Baltimore, MD 21205, USA; 3 Institute of Regional Health Research, University of Southern Denmark, 5000 Odense, Denmark

**Keywords:** suicide, psychiatric inpatients, epidemiology, review

## Abstract

People with mental illness have an increased risk of suicide. The aim of this paper is to provide an overview of suicide risk estimates among psychiatric inpatients based on the body of evidence found in scientific peer-reviewed literature; primarily focusing on the relative risks, rates, time trends, and socio-demographic and clinical risk factors of suicide in psychiatric inpatients. Psychiatric inpatients have a very high risk of suicide relative to the background population, but it remains challenging for clinicians to identify those patients that are most likely to die from suicide during admission. Most studies are based on low power, thus compromising quality and generalisability. The few studies with sufficient statistical power mainly identified non-modifiable risk predictors such as male gender, diagnosis, or recent deliberate self-harm. Also, the predictive value of these predictors is low. It would be of great benefit if future studies would be based on large samples while focusing on modifiable predictors over the course of an admission, such as hopelessness, depressive symptoms, and family/social situations. This would improve our chances of developing better risk assessment tools.

## 1. Introduction

People with mental illness have an elevated risk of dying prematurely from both natural and unnatural causes [1,2], and their average life span is found to be about 15–20 years shorter than that of the general population [3]. The excess mortality in psychiatric patients is partly explained by an increased risk of suicide [4,5,6]. Large national register-based studies have shown that about 44% of all people who die by suicide have been admitted to a psychiatric hospital [7,8]. In the long term, approximately 4%–5% of people admitted to a psychiatric hospital will die by suicide [6]. Psychological autopsy studies have estimated that up to 90% of people who died by suicide met the criteria for a psychiatric disorder [9,10]. Interviews have found that up to two-thirds of people after self-harm indicated that they had attended mental health care prior to the incident [11,12,13]; thus, the association between mental illness and suicidal behaviour is well-established.

The risk of suicide in psychiatric patients varies over time, and an excessive risk of suicide has been found in patients who are currently admitted or who have been recently discharged from a psychiatric hospital [14,15,16,17]. Although suicides among hospitalised psychiatric patients are rare, it is considered to be a public health problem [18]. Given that many are admitted due to suicidal predispositions, it is thus common practice to evaluate patients’ suicide risk. While suicidal ideation in inpatients may in some cases be inevitable, a hospital stay is still considered protective against suicide, and when, despite precautions, suicide deaths occur, it causes immense distress for both family members and ward staff [19,20].

This paper aims to provide an overview of the scientific literature addressing the risk of suicide associated with a psychiatric inpatient stay. We will be addressing the relative risk, rates, time trends, and socio-demographic and clinical risk factors of suicide in psychiatric inpatients and conclude with a discussion and implication of the available evidence based peer-reviewed literature.

## 2. Relative Risk

Approximately 5% of all suicides occur among currently admitted psychiatric patients [21,22,23,24]. Although 5% may sound low, the relative risk of dying by suicide whilst being admitted to psychiatric hospital is alarmingly high compared with the general population. For example, psychiatric inpatients with an affective disorder have an almost 150 times higher suicide risk than people who have never been hospitalised; the excess risk is found across psychiatric diagnoses [21]. Overall, psychiatric inpatients have a rate of 147 suicides per 100,000 patient-years [14], which is almost 13 times higher than the annual global age-standardized suicide rate of 11.4 per 100,000, provided by the World Health Organization (WHO) [25]. While acknowledging that not all people with mental disorders receive treatment, a recent study found that the risk of suicide varies according to the level of mental health care received; people admitted as psychiatric inpatients within the last year had a 44-fold higher risk of suicide than the general population, whereas those receiving outpatient care or psychiatric medication had an 8- or 6-fold higher risk, respectively [15]. It would probably be wrong to conclude that the treatment causally increases risk of suicide; presumably, effective treatment would decrease the risk of suicide compared to a scenario in which that person did not receive treatment. The association is likely explained through selection; people with frequent psychiatric contact might be at higher risk of dying by suicide because their psychiatric symptoms are more severe than those of patients with less frequent contact. As such, the results could indicate that the psychiatric treatment system successfully identifies people who require treatment, implying that people who have been psychiatrically hospitalized constitute an important group for suicide preventive measures.

A large national cohort study from Denmark noted a decreasing suicide rate among psychiatric inpatients towards the end of the last century but at a significantly slower pace compared to the rate of people who had not been admitted to psychiatric hospital [21], thus suggesting that the excess mortality by suicide among psychiatric inpatients had grown over time. However, an update of this study is needed to determine the current trends of suicide among psychiatric inpatients versus the general population.

## 3. Psychiatric Inpatient Suicide: When, Where and How

The risk of suicide is highest in recently admitted patients, and a large nationwide study found that the median admission time of those who died by suicide was 18 days (interquartile range (IQR): 6, 57) [16], which is in agreement with others studies. As many as one quarter of inpatient suicides take place within the first week of admission [16,26,27,28,29]. Two of the largest studies showed that the majority of patients did not die at the hospital ward; Danish and English studies reported respectively that 58% [16] and 74% [27] of suicides occurred outside the ward while the patients were admitted. Comparable figures are reported from Switzerland [30], Austria [31], and Canada [32]. Furthermore, the English study listed that 32% died on the ward, 39% were on agreed leave, and 29% had left the ward without notifying the clinical staff [27]. Those who died by suicide during the first week of admission predominantly died either at the ward or off the ward while absconding. On the other hand, patients who died by suicide after more than a week of admission were more likely to have been on agreed leave. A recent large scale observational study compared patients admitted to hospitals or wards with or without locked doors and found no difference in the risk of completed suicide [33]. Several studies [16,27,31,32] have noted that the most frequently used methods among inpatients are hanging (especially if it happens at the ward) or jumping from a height or in front of a vehicle (especially among absconders) [23], while suicides by overdose and drowning are less frequent.

## 4. Prevalence, Rates and Time Change of Inpatient Suicide

Cohort studies have found that suicide occurs in between 0.14%–0.32% of all psychiatric inpatients, while suicides are found to occur with a frequency of 0.08%–0.19% per admission [16,34,35,36,37].

Table 1 lists inpatient suicide rates with respect to admissions, patients, and inpatient person-years, and when comparing rates it is obviously important that the denominator is the same. Most studies have reported rates of inpatient suicide per number of admissions or patients, probably because this type of data is easier to collect through routine clinical data. Inpatient rates have been estimated to be between 54 and 404 suicides per 100,000 admissions [31,34,36,37,38,39,40,41,42,43] and between 127 and 332 suicides per 100,000 patients [35,37,43,44,45]. Expectedly, rates calculated per inpatient are higher than rates calculated per admission, as one patient can have more than one admission. Only few studies have reported rates calculated per inpatient person-years, i.e., using time at risk (as an inpatient) for the rate estimation. Estimating rates per inpatient person-years is often viewed as more informative as it allows for a comparison with rates of other groups, such as the background population. In this manner, the relatively high rates in psychiatric inpatients can be visualised more clearly. A study from 1980 revealed a suicide rate of 450 per 100,000 inpatient years, thus using time at risk (inpatient person-years) for the suicide rate estimation [34]. A recent English study measured suicide rates according to inpatient person-days and noted a rate of 1.02 per 100,000 total bed days (corresponding to 373 per 100,000 inpatient person-years) [46], and a study from Taiwan showed rates of 686 and 295 per 100,000 inpatient person-years, respectively, for the periods 1985–1995 and 1998–2008. A Danish study reported an even higher rate of 860 per 100,000 inpatient person-years [16]. The severity of inpatient suicides becomes more worrisome when comparing this rate with that of the background population, which was 11.4 per 100,000 person-year [25].

Table 1 illustrates the variability in inpatient suicide rates. This might be related to differences between the included studies with relation to: (1) the psychiatric population admitted to hospital; (2) the level of provided care (e.g. staff per patient); (3) period effects; or (4) level of ‘suicide’ safety measures implemented in the hospitals (some wards might have implemented better restrictive measures for preventing access to suicidal means). A recent meta-analysis by Walsh et al. [14]. on the rates of psychiatric inpatient suicide found that inpatient suicide rates were higher in recent studies compared with studies based on data prior to 1980 [14]. The authors suggested that the observed increase in inpatient suicide rates could be explained by the de-institutionalization processes, leading to a change in the diagnostic profile of patients admitted to hospital. For instance, patients with less severe mental illnesses are nowadays to a larger extent treated in outpatient settings, implying a higher proportion of severely ill patients with high risks of suicide among inpatients. Consequently, a higher suicide rate would be expected among inpatients. While Walsh et al. [14] noted in their meta-analyses that previous studies found lower suicide rates than more recent studies, only few studies have actually addressed the period effects of inpatient suicide rates. Large cohort studies from England, Israel, and Denmark have reported a significant decreasing trend in inpatient suicide rates from the late 1990s into the new millennium [49,50,51]. The English study found that the decrease was highest in the youngest age group (15–44 years) and among patients with schizophrenia, whereas the Danish study noted that the decrease was most pronounced among female patients. Both these studies were conducted during periods of de-institutionalization, and both studies discussed whether these processes, such as shorter inpatient stays, might have resulted in a transfer of the suicide risk from the inpatient period to the discharge period. Both studies found that the suicide rate had changed among those patients recently discharged. In England, the rate of post-discharge suicide rose from 1.39 per 1000 discharges in 1997/98 to 1.66 per 1000 discharges in 2007/08 [50], whereas, in Denmark, the post-discharge suicide rate was found to have decreased [52].

## 5. Predictors of Inpatient Suicide

Several studies have examined the predictors of inpatient suicide in those admitted to a psychiatric hospital. The main findings are summarized in the following two tables; Table 2 lists studies that have examined the predictors for psychiatric inpatient suicide with a control patient population, while Table 3 lists the main findings from these studies.

Table 2 reveals that most studies were based on relatively small samples and lists only few cases of inpatient suicides. Furthermore, most studies used a case-control design and logistic regression analyses. Some few studies were based on patients with a certain diagnosis only [30,35,38,53], or the analysis was conducted separately for different diagnoses [44] (see comments in Table 2). Only one study included a national sample of patients [16]. In other words, Table 2 shows the variation in study designs and power. Table 3 discloses that our knowledge on the socio-demographic and clinical predictors for inpatient suicide, despite many years of research, remains uncertain and limited. For instance, most original studies, as well as the meta-analyses, reported non-significant findings with respect to gender. However, the two largest studies [16,27] (as measured by most cases of suicide) both find that the male sex was associated with a more than twofold higher risk of suicide compared with female inpatients. The results regarding age also point in different directions; some studies found support for a higher risk among younger patients, while others showed that the risk increased with increasing age, and yet others reported no significant impact of age. In the background population, male gender and increasing age are generally associated with a higher risk of suicide in Western countries [59]. Interestingly, a few studies found that higher education, high income, and being employed (as opposed to unemployed) were associated with an increased risk of suicide. Generally, higher education, high income, and being employed have been associated with lower mortality rates, including suicide [59,60]. It has been discussed whether the reverse associations between these social-economic variables and suicide in psychiatric populations might be explained by well-functioning people who suddenly find themselves in a stressful situation when facing psychiatric illness because they: (1) are afraid of losing income, employment, or the ability to use their education; (2) might feel more stigmatized by their social surroundings; or (3) have a greater insight into the course of the mental illness. The last is a commonly held view for which the existing literature presents conflicting evidence as to whether greater insight increases or decreases the risk of suicidal behaviour [44,61,62,63]. The most established predictors of inpatient suicide appear to be depressive symptoms during admission, a diagnosis of an affective disorder or a schizophrenia spectrum disorder, and a history of deliberate self-harm. Evidence for these predictors is supported by a systematic review and meta-analyses by Large et al. [58] (see Table 3). In addition to the predictors listed in Table 3, the meta-analyses identified a family history of suicide or mental illness, high levels of hopelessness, feelings of worthlessness or guilt, prescribed antidepressants, and longer length of hospitalisation as predictors of suicide. No link to suicide was found for social or relationship problems, physical illness, prior criminality/violence, suicidal ideation at admission, agitation or anxiety, delusional ideas, hallucinations, or prescribed antipsychotic drugs. Finally, some studies have suggested that the side effects of psychopharmacological medicine might increase the risk of inpatient suicide [36,40]. However, one of the largest case-control studies [41] of 112 suicides did not find a significant association between medication and suicide. Insights into illness [44] and absconding from hospital [27] have also been associated with risk of suicide.

Also, the comparison across studies is not uncomplicated; several of the studies were based on older psychiatric populations who had received different treatments than more recent populations. Secondly, some studies examined predictors that had not been described in sufficient detail, leaving the reader unable to interpret the findings. For instance, one study examined educational level, broken homes, and living and vocational situations as predictors, but the authors did not describe how these variables were constructed [53]. Several other studies [27,36,40,41,45,57] based on clinical data have only vaguely described how depressive symptoms, hallucinations, or delusions were derived. These are not unitarily defined and could, therefore, be measured differently with respect to the frequency, severity, and type of delusions, making comparisons between studies less reliable. Another issue pertains to the fact that most studies were carried out in high-income countries, and the results might thus not be generalised to other parts of the world.

The identification of predictors of suicide is challenged by the fact that suicide is a rare event, and, when studied in a relatively small population of psychiatric inpatients, it is even more difficult to secure sufficiently robust risk estimates. It is appropriate to mention some of the limitations that apply to several of the presented studies. Numerous studies were based on a few observations of suicide deaths (see Table 2); only a few original studies included more than 100 inpatient suicide deaths [16,27,41]. Consequently, many of the presented estimates were based on univariate associations with no adjustment for confounding variables and wide confidence intervals. On the other hand, some of the studies that presented multivariate associations did not provide the results from the univariate analysis, leaving the reader unable to evaluate whether adjustment lead to changes in the estimates [37,53] (see Table 3). In addition, most studies used a case-control design, in which the ability to generalise the results to the full population of psychiatric inpatients may be compromised because the selection of controls might be biased. For example, some studies matched cases to the next patient admitted to the same hospital or ward [30,39,54], increasing the probability of a comparison to patients frequently hospitalised, potentially representative of patients with a more chronic disease profile.

## 6. Clinical Implication

The aim of this review was to describe the rates and risk factors of inpatient suicides. We have compared inpatients to the background population, examined differences in risk among psychiatric inpatients, and assessed risk predictors for inpatient suicide. Overall, and not surprisingly, the findings from various scientific studies tell us that patients admitted to psychiatric hospitals have an extremely high risk of suicide while being in treatment with compared to the background population. However, what also stands out is that between 0.14%–0.32% of all psychiatric inpatients die by suicide while admitted. In other words, psychiatric inpatients constitute a high-risk group with respect to suicide. However, within this group, it can be difficult to identify the few patients who will die by suicide due to the low prevalence of suicide.

In terms of clinical implications, clinicians should always be alert regarding the suicide risks of psychiatric patients, and this should, ideally, be assessed and addressed. A conversation focusing on suicidal risk with the patient should be carried out in a calm setting where trust and confidence between patient and clinician is paramount. Obviously, the conversation should be performed in a flexible and caring manner. Above we presented the established predictors of inpatient suicides; however, whether a risk assessment based on these risk predictors is valid has been debated [64,65]. It is difficult to predict suicide because it is a rare event, and, as acknowledged by others, the rate of false positives is high; most psychiatric patients will never display suicidal behaviour despite presenting one or several risk predictors [66]. Studies examining the predictive value of risk assessment based on known high-risk predictors have shown a lack of certainty in the prediction. An algorithm for the exact calculation of suicide risk will probably never be achieved, especially since suicidal ideation and symptoms of depression fluctuate, while impulsivity also plays a major role, implying a constant change in suicidal risk. Nevertheless, checklists or risk screening might provide guidance to which items ought to be covered in a conversation focusing on risk assessment.

## 7. Conclusions

In sum, psychiatric inpatients have a high risk of suicide, but it is still a challenge for clinicians to identify those patients that are most likely to die by suicide during admission. This review concluded that the literature on inpatient suicide in most cases was based on studies with low power (few suicide cases), compromising the quality and generalisability of the study results. The few studies with some power mainly identified non-modifiable risk predictors, such as male gender, diagnosis, or recent deliberate self-harm. It would be of great benefit if future studies of inpatient suicide were based on large samples sizes and focused on modifiable predictors that can change over the course of admission, such as hopelessness, depressive symptoms, and family/social situations. This could improve our chances for developing better risk assessment tools. In order to prevent inpatient suicides, a continuous focus on the risk of suicide is necessary, ideally in dialogue with the inpatient. This focus ought to go hand in hand with aims of improving care for people with mental illness, through effective communication between wards, with the aim of improving risk management in inpatient settings, as well as reducing accessibility to the means and possibilities for absconding.

## Figures and Tables

**Table 1:** Rates of inpatient suicide.

Study	Study Period	Suicides/100,000 Admissions	Suicides/100,000 Patients	Suicides/100,000 Inpatient Years
Madsen et al. 2013 [16]	1998–2005	78	221	860
Deisenhammer, 2000 [31]	1987–1994	132		
Gale et al. 1980 [34]	1975–1977	188		450
Krupinski et al. 1998 [35]	1981–1992		324	
Neuner et al. 2008 [36]	1995–2005	101		
Spiessl et al. 2002 [37]	1989–1999	76	142	
Taiminen et al. 1994 [38]	1967–1993	404		
Read et al. 1993 [39]	1984–1989	204		
Dong et al. 2005 [40]	1997–1999	269		
Powell et al. 2000 [41]	1963–1992	137		
Cheng et al. 2009 [42]	1995–2004	162		
Lin et al. 2014 [43]	1998–2008	54	127	295
Lin et al. 2014 [43]	1985–1995	185	332	686
Steblaj et al. 1999 [44]	1984–1993		243	
Li et al. 2008 [45]	1956–2005		133	
Ajdacic-Gross et al. 2009 [47]	1992–2004	161		
Jones et al. 2011 [48]	1972–2000			162
Levi et al. 2016 [49]	1990–2013	75		286
Kapur et al. 2006 [50]	1997–2003			373 *

* Estimated/converted by the authors from 1.02 suicides per 100,000 bed days.

**Table 2:** Studies examining predictors of suicide among psychiatric inpatients.

Author, Year	Suicides/Non Suicides	Design	Statistical Analysis	Comments
Madsen et al. 2012 [16]	279/126,382	Cohort	Cox regression	National sample
Hunt et al. 2007 [27]	222/222	Case-control	Logistic regression	
Modestin et al. 1992 [30]	53/53	Case-control	Logistic regression	Only patients with schizophrenia
Gale et al. 1980 [34]	60/5105	Cohort	χ^2^-test	
Krupinski et al. 1998 [35]	33/3759	Cohort	Discriminant analysis	Patients with affective psychosis
Neuner et al. 2008 [36]	41/20,543	Cohort	Logistic regression	
Spiessl et al. 2002 [37]	30/21,062	Cohort	Logistic regression	
Read et al. 1993 [39]	27/86	Case-control	Logistic regression	
Taiminen et al. 1994 [38]	28/28	Case-control	Logistic regression	Patients with schizophrenia or paranoia
Powell et al. 2000 [41]	112/112	Case-control	Logistic regression	
Lin et al. 2014 [43]	43/162	Case-control	Logistic regression	
Steblaj et al. 1999 [44]	79/79	Case-control	Logistic regression	Analyses carried out in diagnostic groups
Li et al. 2008 [45]	64/64	Case-control	Logistic regression	
Ajdacic-Gross et al. 2009 [47]	141/87,341	Cohort	Poisson regression	
Levi et al. 2016 [49]	326/1304	Case-control	Logistic regression	National sample
Modestin & Kopp, 1988 [53]	75/50	Case-control	Logistic regression	
Roy & Draper, 1995 [54]	37/37	Case-control	χ^2^-test	
Shah & Ganesvaran, 1997 [55]	60/60	Case-control	χ^2^-test, *t*-test	
Sharma et al. 1998 [56]	44/44	Case-control	χ^2^-test, ANOVA	
King et al. 2001 [57]	59/106	Case-control	Logistic regression	
Large et al. 2011 [58]		Systematic review	Meta-analysis Pooled odds ratios	29 case-control studies

ANOVA: Analysis of variance.

**Table 3:** Outline of examined predictors for inpatient suicide. Reported risk estimates are all significant with a *p*-value below 0.05.

Variable (Study)	Risk Estimate Unadjusted	Risk Estimate Adjusted	Comment	Variable (Study)	Risk Estimate Unadjusted	Risk Estimate Adjusted	Comment
***Male sex***				***Age***			
Madsen et al. [16]		2.58					
Hunt et al. [27]	1.49	2.39		Madsen et al. [16]		1.18	With increasing age
Gale et al. [34]	NS			Krupinski et al. [35]	NS		Comparing 10-year age groups
Krupinski et al. [35]	NS			Neuner et al. [36]	(+)	NS	Higher risk among younger patients.
Neuner et al. [36]	NS	NS		Powell et al. [41]	1.8		Risk for patients. <45 years of age
Read et al. [39]	NS			Lin et al. [43]	NS		
Powell et al. [41]	NS	NS		Sharma et al. [56]	NS		Comparing mean age
Li et al. [45]	NS			Large et al. [58]	NS		With increasing age
Ajdacic-Gross et al. [47]		1.75					
Levi et al. [49]	1.85	1.92					
Modestin & Kopp [53]		(+)	Male sex increased risk	***Unemployment***			
Shah & Ganesvaran [55]	NS			Madsen et al. [16]	NS	NS	
				Hunt et al. [27]	0.21	0.17	Compared to employed
Sharma et al. [56]	NS			Spiessl et al. [37]		5.21	OR for part-time employed
Large et al. [58]	NS			Read et al. [39]	NS		
***Educational level***				Dong et al. [40]	NS	NS	
Madsen et al. [16]	2.86	2.44	For bachelor degree and up versus primary school	Powell et al. [41]	NS		
Modestin et al. [30]	NS			Roy & Draper [54]	NS		
				Sharma et al. [56]	NS		
Gale et al. [34]	NS		Males only	Large et al. [58]	NS		
Gale et al. [34]	(+)		Females: Higher education⟹higher suicide risk	***Low income***			
Powell et al. [41]	NS	NS	No educational qualifications	Madsen et al. [16]	NS	NS	
Modestin & Kopp [53]		NS		Modestin et al. [30]	(+)		Low social class increased risk
Sharma et al. [56]	NS			Shah & Ganesvaran. [55]	(+)		High social class increased risk
***Marriage/cohabiting***				***Living alone***			
Madsen et al. [16]	0.61	NS	Risk for divorced, widowed	Madsen et al. [16]	0.63	NS	
Hunt et al. [27]	NS	NS		Hunt et al. [27]	NS	NS	
Modestin et al. [30]	NS			Taiminen et al. [38]	NS	NS	
Gale et al. [34]	NS			Dong et al. [40]	NS	NS	
Krupinski et al. [35]	NS			Powell et al. [41]	NS		
Read et al. [39]	4.1		Risk for single, divorced, widowed	Roy & Draper [54]	NS		
Dong et al. [40]	NS	NS		Sharma et al. [56]	NS		
Powell et al. [41]	NS			Large et al. [58]	NS		
Ajdacic-Gross et al. [47]		1.70		***Outpatient treatment***			
Modestin & Kopp [53]		NS		Madsen et al. [16]	NS	NS	
Roy & Draper [54]	NS			Neuner et al. [36]		4.11	Supportive psychotherapy before admission
Sharma et al. [56]	NS						
Large et al. [58]	NS			***Schizophrenia diagnosis***			
***Affective diagnosis***				Madsen et al. [16]	0.44	0.53	Versus affective diagnosis
Madsen et al. [16]	2.27	1.88	Versus schizophrenia diagnosis	Gale et al. [34]	(+)		Increased risk if schizophrenia disorder
Hunt et al. [27]	1.97	1.81	Versus other diagnoses	Spiessl et al. [37]		8.42	
Gale et al. [34]	(+)		Increased risk if affective disorder	Read et al. [39]	5.6		
Powell et al. [41]	NS	NS		Powell et al. [41]	NS	NS	Previous diagnosis of schizophrenia
Levi et al. [49]	6.16	5.95		Lin et al. [43]	0.30	0.42	Psychotic symptoms versus. no psychotic symptoms
Roy & Draper [54]	NS			Levi et al. [49]	5.66	3.82	
Large et al. [58]	1.93	1.93		Roy & Draper [54]	(+)		Increased risk if schizophrenia disorder
***Comorbid substance abuse***				Shah et al. [55]	(+)		Increased risk if schizophrenia disorder
Madsen et al. [16]	NS	NS	Secondary diagnosis	Large et al. [58]	2.48	1.65	
Hunt et al. [27]	NS	NS	History of substance misuse	***Comorbid personality disorder***			
Krupinski et al. [35]	NS		Addiction to alcohol or drugs	Madsen et al. [16]	1.72	1.60	Comorbid personality disorder
Read et al. [39]	NS		History of alcohol/drug abuse	Hunt et al. [27]	1.58	NS	Any secondary psychiatric diagnosis
Dong et al. [40]	NS	NS	History of substance misuse	Powell et al. [41]	NS	NS	Previous personality disorder
Powell et al. [41]	0.2	NS	Current drug abuse	King et al. [57]	NS	NS	Additional axis II disorder
Powell et al. [41]	0.2	0.2	Current alcohol abuse				
Levi et al. [49]	1.67	NS	Abuse at admission	***Planned admission***			
Roy & Draper [54]	NS		Alcohol/drug abuse	King et al. [57]	NS	NS	
King et al. [57]	NS	NS	History of problem drinking				
King et al. [57]	NS	NS	History of substance misuse				
Large et al. [58]	NS						
***Depressive symptoms***				***Compulsory admission***			
Hunt et al. [27]	1.60	NS	At last contact	Madsen et al. [16]	NS	NS	
Dong et al. [40]	(+)	NS	(+) depr.sympt. adm. increased risk	Hunt et al. [27]	0.43	NS	
Dong et al. [40]		8.53	At time of suicide	Gale et al. [34]	(+)		Increased the suicide risk
Taiminen et al. [38]		8.55	Depressive symptoms at admission	Krupinski et al. [35]	NS		
Powell et al. [41]	3.1	NS	Depressed mood	Read et al. [39]	5.5		
Lin et al. [43]	4.91	2.11		Dong et al. [40]	NS	NS	
Steblaj et al. [44]		11.94	For pt. with schizophrenia	Powell et al. [41]	NS	NS	Previously detained under Mental Health Act
Steblaj et al. [44]		38.47	For pt. with affective psych.osis	Roy & Draper [54]	(+)		Increased the suicide risk
King et al. [57]	2.28	23.45	Depressive symptoms	King et al. [57]	3.27	49.83	Wide CI for adjusted estimate
Large et al. [58]	3.92	2.34	Depressed mood at admission	Large et al. [58]	1.87	NS	
***Recent DSH***				***Psychiatric history***			
Madsen et al. [16]	4.05	4.99		Hunt et al. [27]	NS	NS	Duration of illness <12 month
Hunt et al. [27]	3.31	NS		Hunt et al. [27]	NS	NS	>5 previous admissions
Modestin et al. [30]	(+)	(+)	DSH increased risk	Modestin et al. [30]	(+)	NS	Mental illness >5 years
Spiessl et al. [37]		NS		Krupinski et al. [35]	NS		Last admission within last year
Dong et al. [40]		3.92		Taiminen et al. [38]	NS	NS	Total No. of admissions
Powell et al. [41]	14.3			Read et al. [39]	NS		Prior psychiatric admissions
Li et al. [45]	5.17			Dong et al. [40]	NS	NS	Duration of illness <12 month
Levi et al. [49]	4.10	2.59		Powell et al. [41]	2.3	2.5	One or more previous admissions
Modestin & Kopp [53]		(+)	DSH increased risk	Powell et al. [41]	2.7	2.9	Chronic mental ill (>5 years)
Shah et al. [55]	(+)		DSH increased risk	Steblaj et al. [44]	NS		Mean duration of illness
King et al. [57]	3.95	1.26		Li et al. [45]	(+)		Suicide associated with more previous admissions
Large et al. [58]	2.41	2.26		Levi et al. [49]	1.31	1.16	No. of hospitalizations
				Modestin & Kopp [53]		(+)	Mean Number of previous admissions. increased risk
				Modestin & Kopp [53]		NS	Duration of psychiatric illness
***History of DSH***				Sharma et al. [56]	NS		Mean no. of previous admissions
Madsen et al. [16]	1.36	1.91		King et al. [57]	NS	NS	Length of psychiatric illness
Hunt et al. [27]	4.30	5.93		King et al. [57]	NS	NS	Previous admissions (0 vs. 1 vs. >1)
Modestin et al. [30]	(+)	NS	(+) DSH increased risk	Large et al. [58]	1.81	1.58	Median No. of admissions
Krupinski et al. [35]	(+)		DSH increased risk				
Neuner et al. [36]		4.34					
Spiessl et al. [37]		5.6					
Taiminen et al. [38]	(+)		DSH increased risk				
Read et al. [39]	3.6						
Dong et al. [40]		4.60					
Powell et al. [41]	3.4	2.2	Only significant for patients with schizophrenia				
Modestin & Kopp [53]		NS					
Roy & Draper [54]	(+)		DSH increased risk				
Shah et al. [55]	(+)		DSH increased risk				
Sharma et al. [56]	(+)		DSH increased risk				
King et al. [57]	2.23	NS					
Large et al. [58]	3.95	3.30					

CI: confidence interval; DSH: deliberate self-harm; NS: not statistically significant; OR: odds ratio; (+): statistically significant at the 0.05 level, no estimate reported, but direction of association described in Comment section.
